# Creating mosquito-free outdoor spaces using transfluthrin-treated chairs and ribbons

**DOI:** 10.1186/s12936-020-03180-1

**Published:** 2020-03-10

**Authors:** John P. Masalu, Marceline Finda, Gerry F. Killeen, Halfan S. Ngowo, Polius G. Pinda, Fredros O. Okumu

**Affiliations:** 1 Environmental Health and Ecological Sciences Department, Ifakara Health Institute, Morogoro, United Republic of Tanzania; 2 School of Life Science and Biotechnology, Nelson Mandela African Institution of Science and Technology, Arusha, United Republic of Tanzania; 3 School of Public Health, University of the Witwatersrand, Parktown, Johannesburg, Republic of South Africa; 4 Department of Vector Biology, Liverpool School of Tropical Medicine, Liverpool, UK; 5 Institute of Biodiversity, Animal Health and Comparative Medicine, University of Glasgow, Glasgow, UK

**Keywords:** Peri-domestic spaces, Transfluthrin-treated chairs, Eave ribbons, Transfluthrin, Spatial repellents, Outdoor-biting, Malaria vectors, Ifakara Health Institute

## Abstract

**Background:**

Residents of malaria-endemic communities spend several hours outdoors performing different activities, e.g. cooking, story-telling or eating, thereby exposing themselves to potentially-infectious mosquitoes. This compromises effectiveness of indoor interventions, notably long-lasting insecticide-treated nets (LLINs) and indoor residual spraying (IRS). This study characterized common peri-domestic spaces in rural south-eastern Tanzania, and assessed protective efficacy against mosquitoes of hessian fabric mats and ribbons treated with the spatial repellent, transfluthrin, and fitted to chairs and outdoor kitchens, respectively.

**Methods:**

Two hundred households were surveyed, and their most-used peri-domestic spaces physically characterized. Protective efficacies of locally-made transfluthrin-emanating chairs and hessian ribbons were tested in outdoor environments of 28 households in dry and wet seasons, using volunteer-occupied exposure-free double net traps. CDC light traps were used to estimate host-seeking mosquito densities within open-structure outdoor kitchens. Field-collected *Anopheles arabiensis* and *Anopheles funestus* mosquitoes were exposed underneath the chairs to estimate 24 h-mortality. Finally, The World Health Organization insecticide susceptibility tests were conducted on wild-caught *Anopheles* from the villages.

**Results:**

Approximately half (52%) of houses had verandas. Aside from these verandas, most houses also had peri-domestic spaces where residents stayed most times (67% of houses with verandas and 94% of non-veranda houses). Two-thirds of these spaces were sited under trees, and only one third (34.4%) were built-up. The outdoor structures were usually makeshift kitchens having roofs and partial walls. Transfluthrin-treated chairs reduced outdoor-biting *An. arabiensis* densities by 70–85%, while transfluthrin-treated hessian ribbons fitted to the outdoor kitchens caused 77–81% reduction in the general peri-domestic area. Almost all the field-collected *An. arabiensis* (99.4%) and *An. funestus* (100%) exposed under transfluthrin-treated chairs died. The *An. arabiensis* were susceptible to non-pyrethroids (pirimiphos methyl and bendiocarb), but resistant to pyrethroids commonly used on LLINs (deltamethrin and permethrin).

**Conclusion:**

Most houses had actively-used peri-domestic outdoor spaces where exposure to mosquitoes occurred. The transfluthrin-treated chairs and ribbons reduced outdoor-biting malaria vectors in these peri-domestic spaces, and also elicited significant mortality among pyrethroid-resistant field-caught malaria vectors. These two new prototype formats for transfluthrin emanators, if developed further, may constitute new options for complementing LLINs and IRS with outdoor protection against malaria and other mosquito-borne pathogens in areas where peri-domestic human activities are common.

## Background

Since 2000, malaria morbidity and mortality have tremendously declined in sub-Saharan Africa [1–4], though the recent evidence suggests that such gains are starting to stagnate [3–5]. Most of the gains observed between 2000 and 2015 were estimated to have been contributed by the existing core indoor vector control interventions, i.e. insecticide-treated nets (ITNs) and indoor residual spraying (IRS) [2, 6–8]. Long-lasting insecticide-treated nets (LLINs) and IRS are effective against indoor-biting and indoor-resting mosquitoes, but are less effective against outdoor-biting mosquitoes, which are important vectors of residual malaria transmission [9–12]. It has been estimated that the *Anopheles* bites not preventable by LLINs could be causing up to 10 million additional malaria cases annually [12]. As a result, LLINs and IRS require complimentary interventions to achieve the 2030 global targets of reducing malaria burden by at least 90% and elimination in 35 endemic countries [13].

In many malaria-endemic communities, people spend several hours cooking, eating and socializing outdoors in the early evenings before they go to sleep, and also in the early mornings after they wake up [14], when malaria vectors may be active and mediating transmission [11]. Some of these outdoor activities, as well as sleeping outdoors [15], are partly attributable to warm climate [16], but they also have strong cultural determinants [17]. The importance of outdoor malaria transmission, and associated outdoor human activities, are now well-established [9, 10, 14, 17]. However, there are still gaps regarding appropriate interventions to address these gaps. The characteristics of the peri-domestic spaces where households conduct outdoor activities remain poorly documented, despite being essential for designing, creating and testing interventions to complement LLINs and IRS by protecting such outdoor spaces.

Several intervention options have been proposed as candidates for closing these malaria transmission gaps [18]. Examples include: (a) outdoor-baited traps [19, 20], (b) attractive targeted sugar baits [21], (c) pyrethroid-treated clothing [22, 23], zooprophylaxis [24] and repellents [25] among others. Topical repellents applied on human skin are widely available for personal protection in some areas. However, commercial formulations of government-sectioned scale-up campaigns of such topical repellents are limited because they protect only individual users [26], have low user compliance rates and acceptance [27–29], and have only short-term efficacy [30]. They are also expensive for repeated use by the low-income populations at greatest risk.

In contrast, spatial repellents are volatile insecticides that diffuse into the air as vapour, and may protect multiple people within the surrounding space against outdoor-biting malaria vectors [31–35]. In recent years, several versions and delivery formats have been developed, which allow wide-area protection of multiple persons without repeated application for several months [31–34, 36, 37]. In particular, a wide range of transfluthrin emanator prototypes based on treated hessian fabric products have been recently developed that protect indoor and outdoor spaces for several months without repeated reapplication [31–34, 36, 37]. Transfluthrin also has additional properties beyond just spatial repellency that include toxicity to mosquitoes, and incapacitation that prevents blood-feeding, which could contribute to community-wide mass effects, even for non-users [37, 38].

Improved understanding of the peri-domestic spaces coupled with new interventions that can be effective in such spaces, could potentially address current challenges related with exposure to outdoor-biting exposure and transmission risk. This study was, therefore, aimed at addressing two key knowledge gaps by: (a) characterizing the common peri-domestic spaces used by communities in rural south-eastern Tanzania for various outdoor activities, and, (b) assessing the protective efficacies of two recently-developed hessian-based transfluthrin-emanator prototypes, specifically transfluthrin-treated chairs and transfluthrin-treated hessian ribbons wrapped around outdoor kitchens, against outdoor-biting malaria vectors and other pathogens-carrying mosquitoes in those peri-domestic spaces.

## Methods

### Study area

**Fig. 1:**
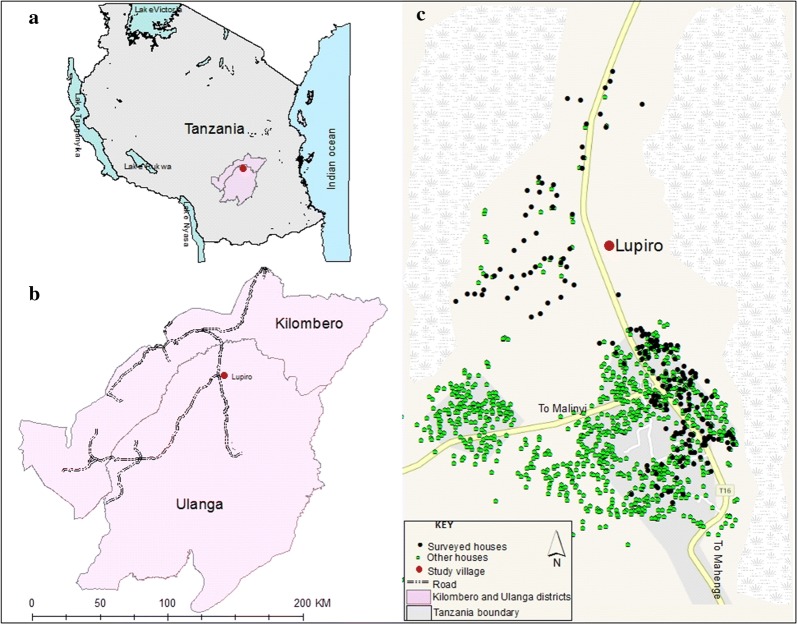
Illustration of the location of Ulanga and Kilombero districts in the map of Tanzania (**a**), the location of Lupiro village in Ulanga district (**b**) and household location in Lupiro village showing both surveyed and those did not (**c**)

The study was implemented in Lupiro village (8.385° S, 36.670° E) (Fig. 1), in the Kilombero valley, south-eastern Tanzania. Households were selected from four sub-villages namely: (a) Ndoro; (b) Libaratula; (c) Mabatini and (d) Lupiro Kati. Most residents here were peasants, cultivating rice, maize and other crops. Houses have brick or mud walls, and metal (corrugated iron sheets) or grass-thatched roofs. Annual rainfall is 1200–1600 mm, and temperatures range between 20.0 and 32.6 °C [39, 40]. Principal malaria vectors in this area are *An*o*pheles funestus* and *Anopheles arabiensis* with the former contributing over 80% of transmission [41]. Both *An. arabiensis* and *An*. *funestus* populations in the area have been shown to be resistant to multiple public health insecticides including pyrethroids, carbamates and organochlorides [41–43]. LLINs are the main malaria prevention method, most of which are distributed by the government [44].

### Characterization of the peri-domestic spaces

Two hundred (200) households were surveyed, including 50 from each sub-village (Fig. 1), selected via stratified random sampling. Data were collected using electronic tablets using *KoboCollect™*, an open access software programmed using Open Data Kit (ODK) [45]. Trained research teams were assigned to each sub-village. Written informed consent was obtained from each of the 200 households. For each household, the peri-domestic spaces were observed directly to characterize them physically based on use, physical site and whether they were built-up or not. Digital pictures were taken of the different peri-domestic environments. The research team also administered survey questions to the household heads to capture: (a) identification information such as age, (b) education level, (c) socio-economic data including source of income, possession of radio, television, cell phone among others, (d) information on peri-domestic spaces such as presence of other peridomestic spaces apart from veranda, and (e) their usage, presence of peri-domestic spaces if the house had no veranda and their usage.

The peri-domestic spaces were classified as either: (a) built-up spaces attached to the main houses, i.e. veranda extensions; (b) built-up spaces not attached to the main houses, e.g. separate kitchens, and (c) non-built-up or other peri-domestic spaces commonly used for various outdoor activities. The outdoor built up structures were also characterized based on the roofing and wall types.

### Transfluthrin-treated chairs and hessian ribbons

**Fig. 2:**
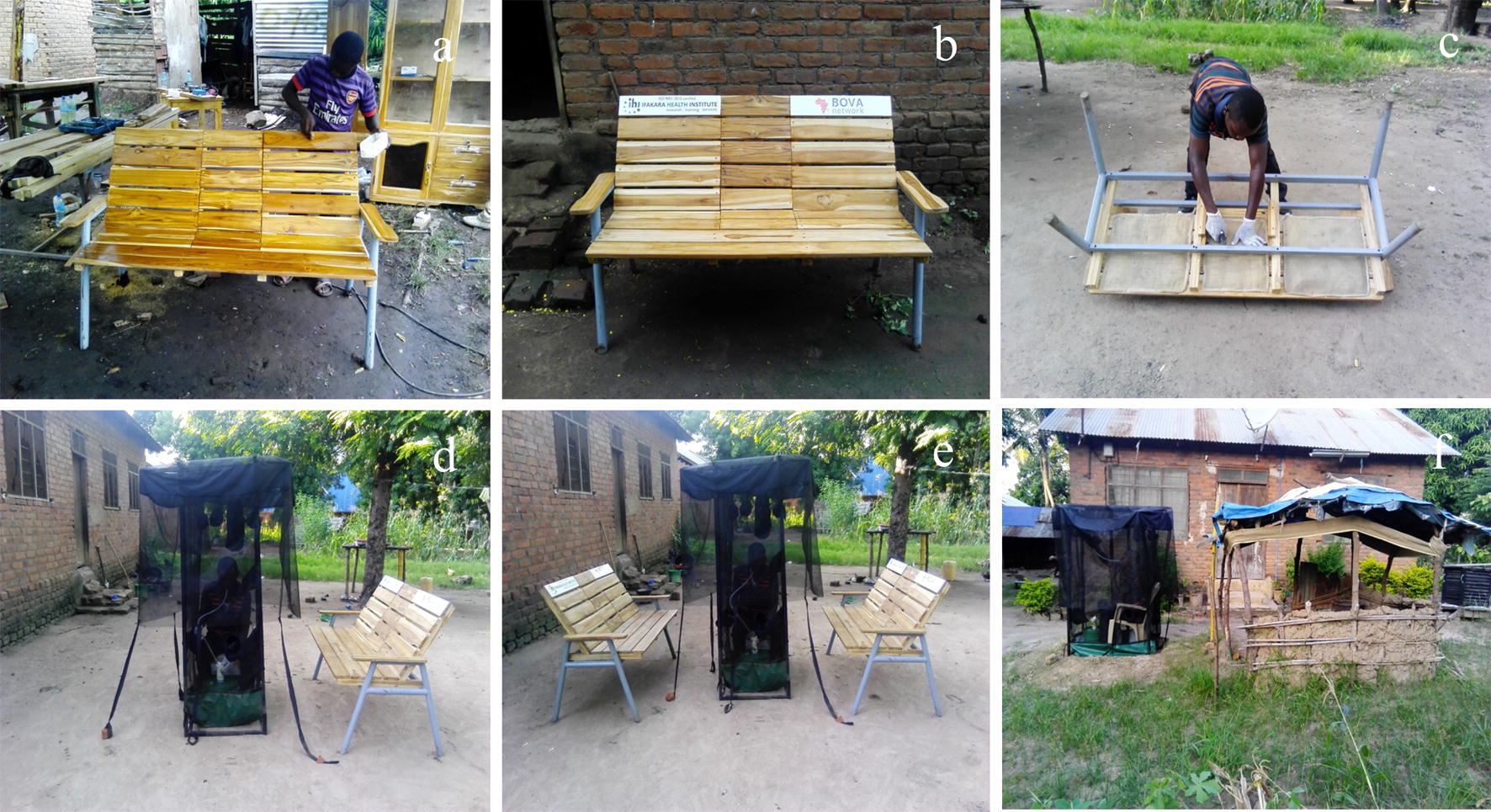
Design and prototyping of the wooden chairs at the local carpentry (**a**), overview of the prototyped chair (**b**), fitting transfluthrin-treated hessian mat underneath the chair (**c**), one transfluthrin-treated chair with the DN-Mini trap positioned 0.5 m (**d**), two transfluthrin-treated chairs with DN-Mini trap installed 0.5 m (**e**); and outdoor kitchen fitted with transfluthrin-treated sisal ribbon with DM-Mini trap positioned 1.2 m (**f**)

For the dry season experiment, six identical chairs made of wood and metal frame were constructed by a local carpenter while for the wet season experiment 15 chairs were made (Fig. 2a, b). The chairs were fitted underneath with four standardized hessian fabric mats: two measuring 42 cm × 43 cm and fitted underneath the right and left sides of the chair and other two measuring 20 cm × 33 cm, which were fitted underneath the middle part of the chair (Fig. 2c). These mats were made by a local seamstress at the Ifakara Health Institute fabrication facility (the MozzieHouse). The hessian mats had been treated in emulsified solutions containing 2% transfluthrin (Bayer AG, Germany), prepared as previously described [31, 33].

Similarly, the hessian ribbons were prepared as previously described by Mmbando et al. [36]. Each ribbon had 15 cm width and 10 m length, and were also made locally at the MozzieHouse. More detailed descriptions of the hessian ribbons have previously been published by Ogoma et al. [31] and Mmbando et al. [36]. The ribbons were also treated in a 2% emulsified solution of transfluthrin as previously described [36].

### Assessing protective efficacies of transfluthrin-treated chairs and ribbons

This assessment was conducted in two seasons: dry and wet seasons, between September to October 2019 and between January to February 2020 as dry and wet seasons, respectively. Following the characterization of the peri-domestic spaces as described above, eight households with outdoor kitchens were selected for a small-scale assessment of protective efficacies of the two candidate interventions in the dry season. The houses were paired and assigned as follows: (a) a control arm, where neither transfluthrin-treated chairs nor transfluthrin-treated ribbons were used, (b) a treatment arm where one transfluthrin-treated chair was used, (c) a second treatment arm where two transfluthrin-treated chairs were used, and (d) a third treatment arm where transfluthrin-treated hessian ribbons were used around the outdoor kitchens. In each arm, two houses were enrolled.

One consenting adult male volunteer was assigned to each household, to sit inside the exposure-free miniaturized double nets trap (DN-Mini) [46] from 1900 to 2300 h. The volunteer spent 45 min each hour retrieving all host-seeking mosquitoes caught in the DN-Mini while attempting to bite him. For the households with transfluthrin-emanating chairs, the DN-Mini was installed 0.5 m from the chairs (Fig. 2d, e). For households with transfluthrin-treated hessian ribbons, the ribbon was fitted 1.3 m above ground (Fig. 2f) onto the outdoor kitchens. CDC light traps [47] were suspended inside these makeshift kitchens to collect host-seeking mosquitoes nightly, while DN-Mini traps were set beside the kitchens to assess biting risk in the general peri-domestic space (Fig. 2f).

Each treatment arm was initially located in two houses per experimental night, but was rotated between the houses using a 4 × 4 Latin square design over 32 experimental nights, so that each treatment or control arm was tested at each of the eight houses four times. The primary outcome was number of mosquitoes of different species caught in the DN-Mini or the CDC light traps per house per night. All treated materials were carefully shifted between the houses to avoid any contamination during the rotations. As the experiments were conducted outdoors with enough airflow, there was no need to break for wash out. Instead, a control set up was used to monitor mortality of mosquitoes as described in the sub-section below. Each morning the collected mosquitoes were sorted and identified using morphological keys [48]. In the wet season, 20 households were enrolled making five households in each arm for other 32 nights. The same procedure was adopted as described in dry season.

### Assessing mortality effects of the transfluthrin-treated chairs on mosquitoes

This assay was done using three different groups of mosquitoes, as follows: (a) field-collected *An. arabiensis* and *An. funestus* of unknown age, which are known to be pyrethroid resistant in this setting [41–43], (b) laboratory-reared *An. arabiensis* from a pyrethroid-susceptible colony of local origin, and (c) laboratory-reared *Aedes aegypti* from a pyrethroid-susceptible colony of local origin [49].

The wild-caught *An. arabiensis* females were collected using a separate set of eight DN-Mini traps [46] set outdoors at households without any transfluthrin treatments. Eight consenting adult male volunteers were involved in these collections each night from 1900 h to 0100 h. As population densities of *An. funestus* in this study area were very low, CDC light traps were used to collect adult females of this species from another village (Tulizamoyo (− 8.3669, 36.7336)) approximately 30 km away.

Each morning captured mosquitoes were sorted and *An. arabiensis* and *An. funestus* females separated in two cages containing 100 mosquitoes per species (four cages in total). Since the *Anopheles gambiae* sensu lato (s.l.) in this area are known to consist exclusively of *An. arabiensis* [33], no molecular identification was required. Similarly, since indoor collections of *An. funestus* s.l. have consistently been found to be > 90% *An. funestus* sensu stricto [50], it was assumed that these were the dominant species in the collections. The separated mosquitoes were kept at a field insectary (average temperature: 26.75 ± 0.09 °C; relative humidity: 73.26 ± 0.46%) for acclimatization for at least 20 h before testing the next evening.

For the tests, two chairs were placed within open verandas of two separate houses. One of the chairs was fitted underneath with transfluthrin-treated hessian mats, while the other was fitted with an untreated hessian mat (control). The caged mosquitoes were placed underneath each chair overnight (1900 h to 0700 h). A simple water moat was used to prevent ants from eating the mosquitoes. Each morning, the cages were returned to the field insectary and monitored for further 12 h, totaling 24 h of observation since start of exposure. This procedure was repeated 10 times (totaling 1140 mosquitoes) for field-collected *An. arabiensis* and five times (totaling 490 mosquitoes) for field-collected *An. funestus* tested in control and treated arms.

Similar tests were conducted using cages containing 100 laboratory-reared *An. arabiensis* or 100 laboratory-reared *Ae. aegypti.* Since *Ae. aegypti* mosquitoes are active during the day, they were exposed from 0800 to 1900 h each day, as opposed to the *Anopheles* mosquitoes, which were exposed at night. Percentage mortality of mosquitoes was calculated for each species separately as a proportion of total exposed.

### Testing susceptibility of local malaria vector populations to common public health pesticides

In order to determine phenotypic resistance status of local mosquito populations to common pesticides, standard discriminatory tests were performed using standard WHO susceptibility bioassays [51]. Since transfluthrin is a pyrethroid, the tests also provided indication of how the transfluthrin-based interventions evaluated here (transfluthrin-treated chairs and transfluthrin-treated hessian ribbons) evaluated here would perform against wild pyrethroid-resistant mosquito populations. The susceptibility tests were done for: (a) 0.1% bendiocarb, a carbamate; (b) 4.0% dichlorodiphenyltrichloroethane (DDT), an organochloride; (c) 0.25% pirimiphos methyl, an organophosphate, (d) 0.75% permethrin, a type I pyrethroid; and (e) 0.05% deltamethrin, a type II pyrethroid.

Female *An. arabiensis* mosquitoes were collected from nearby rice fields as larvae, and reared to emergence at Ifakara Health Institute vector biology laboratory, the VectorSphere. The susceptibility tests were done using 3-day old adult females, using at least 100 mosquitoes per test (25 per replicate), with at least 4 replicates as described in the recent WHO guidelines [51].

### Data analysis

The survey data was summarized in ODK analysis module [45] to generate descriptive statistics of peri-domestic spaces and their usage. Data on efficacy of the transfluthrin-treated chairs and ribbons was analysed using R open-source statistical software [52], primarily using generalized linear mixed-effects models [53], each time modelling the numbers of mosquitoes of a given species caught as a function of the treatments (fixed factors), and fitting the data onto Poisson distributions. Volunteer ID, day and house ID were included as random factors in the models.

## Results

### Characteristics of households

The demographic characteristics of household heads, and physical characteristics of all the 200 houses visited are summarized in Table 1. Most of the household heads were female (128/200). The main construction materials were bricks for the walls (153/200) and corrugated iron sheets for the roofs (140/200). Full details are found in Table 1.

**Table 1:** Characteristics of the study participants and their houses in 200 surveyed households in Lupiro village, Ulanga District, south-eastern Tanzania

Characteristics	Category	Total number surveyed (*n*)	Proportion (%)
Gender	Male	72	36.0
	Female	128	64.0
Age	Average	38.5	NA
Wall type	Bricks	153	76.5
	Mud and stick	46	23.0
	Others	1	0.5
Roof type	Iron-sheets	140	70
	Thatched	56	28.0
	Others	4	2.0

### Characteristics of the peri-domestic spaces

**Fig. 3:**
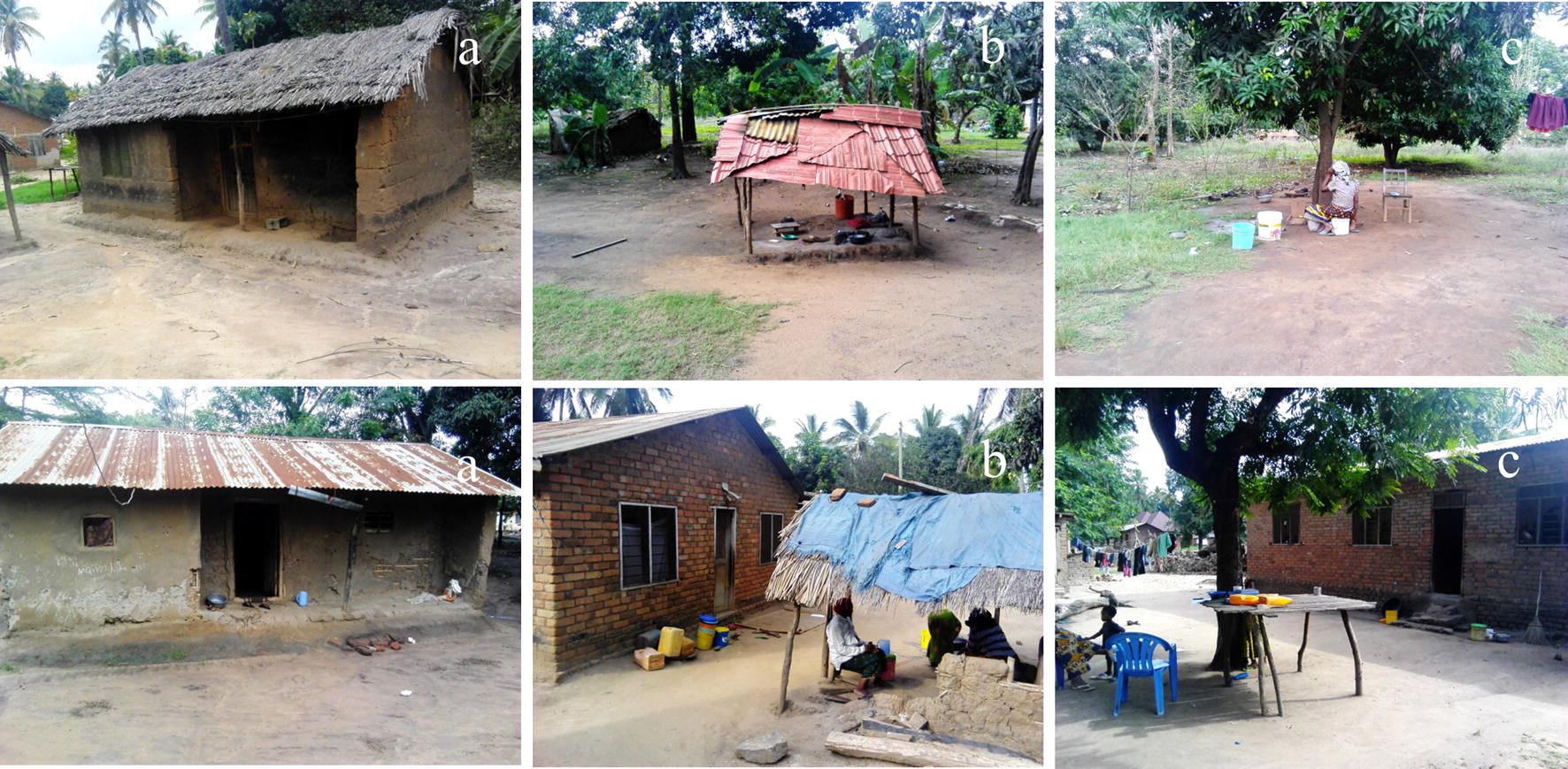
Illustration of houses with veranda extension physically characterized during survey (**a**), houses with built-up peridomestic space away from the main house commonly used for cooking (**b**) and houses with non-built-up peridomestic space physically characterized as under the tree (**c**)

Table 2 provides a summary of the physical characteristics of peri-domestic spaces where residents spent time outdoors in the evenings before bedtime. Of the 200 households observed, 52% (103/200) had built-up veranda (Fig. 3a), while 48% (97/200) did not have these verandas.

**Table 2:** Peridomestic space characteristic of the households surveyed in Lupiro village, Ulanga district, south-eastern Tanzania

Household with veranda (N = 103)			Household without veranda (N = 97)		
Characterization	n	Percentage	Characterization	n	Percentage
Open veranda	69	67.0	N/A		
Closed veranda	34	33.0	N/A		
Usage					
Resting	92	42.2	N/A		
Cooking	67	30.7	N/A		
Eating	56	25.7	N/A		
Others	3	1.4	N/A		
Other peri-domestic space			Other peri-domestic space		
Yes	69	67	Yes	91	93.8
No	34	33	No	6	6.2
Built structure	23		Built structure	32	
Roof	23	100	Roof	31	96.9
No roof	0	0	No roof	1	3.1
Wall	7	30.4	Wall	10	31.3
No wall	16	69.6	No wall	22	68.7
Average distance from the houses (m)	6.3		Average distance from the houses (m)	6.8	
Usage			Usage		
Resting	9	24.3	Resting	19	29.2
Cooking	22	59.5	Cooking	30	46.2
Eating	6	16.2	Eating	16	24.6
Non-built structure	46		Non-built structure	59	
Under the tree	34	62.9	Under the tree	28	42.4
Open space	19	35.2	Open space	34	51.5
Others	1	1.9	Others	4	6.1
Average distance from the houses (m)	6.8		Average distance from the houses (m)	6.2	
Usage			Usage		
Resting	32	43.2	Resting	54	38.0
Cooking	20	27.0	Cooking	48	33.8
Eating	22	29.7	Eating	40	28.2

*n* total number of peridomestic space characterized, *N/A* not required

It was also observed that other than these verandas (Fig. 3a), most houses had additional peri-domestic spaces where members congregated. Of the 103 that had verandas, 69 (67%) also had other active peri-domestic spaces, of which 23 were built-up structures and 46, were non-built up. These structures all had at least physical roofing, and 70% of them also had no wall. Two thirds of the built-up structures were used as outdoor kitchens (60% used for cooking) as shown in Fig. 3b. Many of the non-built structures (63%) were sited under trees (Fig. 3c), while 35% were in open spaces. The peri-domestic spaces were used for multiple activities, e.g. cooking, eating, socializing among others.

Of 97 houses that did not have veranda extensions, 91 (93.8%) had active peri-domestic spaces, of which 32 had built up structures with roofs, and also walls in one-third of the cases. Of the non-built structures, 42% were under trees. Common uses of these spaces were similar, i.e. resting, cooking, eating.

### Overall collected mosquitoes

In the dry season, the total number of mosquitoes collected was 4960, including 2604 *Culex* spp.; 2264 *Anopheles gambiae* s.l.; 80 *Anopheles coustani*; 6 *An. funestus*; 4 *Mansonia* spp.; and 2 *Coquilettidia* mosquitoes. Polymererase chain reaction (PCR) was conducted on 81 samples of *An. gambiae* s.l. to distinguish between sibling species. Of the 90.1% (73/81) successfully amplified in the PCR assays, all (100%) were identified as *An. arabiensis.* In the wet season the total number of mosquitoes collected was 14,303, including 12,224 *Culex* spp.; 1978 *An. gambiae* s.l.; 42 *An. funestus*; 37 *Mansonia* spp.; 15 *Ae. aegypti*; 6 *An. coustani*; and 1 *Anopheles pharoensis*. No molecular assay was conducted to identify mosquito species in this particular season.

### Efficacy of transfluthrin-treated chairs and transfluthrin-treated hessian ribbons against outdoor-biting mosquitoes in the peri-domestic spaces

Findings on protective efficacy of the two interventions are summarized in Tables 3 and 4. Using two transfluthrin-treated chairs significantly reduced outdoor-biting *An. arabiensis* mosquitoes by 76% (Relative rate (RR) = 0.24, 95% confidence interval, CI 0.19–0.29, *P *< 0.001) and by 85% (RR = 0.15, 95% CI 0.12–0.18, *P *< 0.001) in dry and wet seasons, respectively. Using one transfluthrin-treated chair also significantly reduced *An. arabiensis* mosquitoes, in this case by 70% (RR = 0.30, 95% CI 0.25–0.37, *P *< 0.001) and by 75% (RR = 0.25, 95% CI 0.20–0.31, *P *< 0.001) in dry and wet seasons. When the densities of *Culex* mosquitoes were assessed, both the two-chair and one-chair interventions significantly reduced outdoor-biting, achieving 52% (RR = 0.48, CI 0.37–0.63, *P *< 0.001) and 58% (RR = 0.42, 95% CI 0.31–0.56, *P *< 0.001) protection, in dry and wet seasons, respectively. In the wet season, both the two-chair and one-chair interventions significantly reduced outdoor-biting, achieving 51% (RR = 0.49, CI 0.43–0.56, *P *< 0.001) and 40% (RR = 0.60, 95% CI 0.53–0.68, *P *< 0.001) protection.

**Table 3:** Comparison of nightly outdoor biting per person between houses with or without transfluthrin-treated chairs or ribbons (dry season)

Settings	Species	Treatment	Nights	n	Adjusted-mean (95% CI)	RR (95% CI)	PP (95% CI)	P-value
Outdoor peri-domestic space	*Anopheles arabiensis*	Control	32	1056	15.05 (12.29–18.44)	1	0	
		Two TF-chairs	32	273	3.61 (2.87–4.55)	0.24 (0.19–0.29)	0.76 (071–0.80)	< 0.001
		TF-treated ribbon	32	211	2.96 (2.33–3.75)	0.19 (0.16–0.24)	0.81 (0.75–0.84)	< 0.001
		Control	28	910	14.86 (12.07–18.30)	1	0	
		One TF-treated chair	28	290	4.54 (3.60–5.73)	0.30 (0.25–0.37)	0.70 (0.62–0.75)	< 0.001
	*Culex* spp.	Control	32	889	10.52 (7.98–13.86)	1	0	
		Two TF-chairs	32	426	5.12 (3.84–6.83)	0.48 (0.37–0.63)	0.52 (0.36–0.63)	< 0.001
		TF-treated ribbon	32	299	3.43 (2.55–4.61)	0.32 (0.24–0.43)	0.68 (0.57–0.75)	< 0.001
		Control	28	744	9.99 (7.43–13.44)	1	0	
		One TF-treated chair	28	335	4.20 (3.07–5.75)	0.42 (0.31–0.56)	0.58 (0.43–0.68)	< 0.001
Inside outdoor kitchen enclosure	*Anopheles arabiensis*	Control	25	152	1.17 (0.56–2.44)	1		
		TF-sisal ribbon	25	113	0.56 (0.26–1.22)	0.57 (0.32–1.03)	0.43 (− 0.03 to 0.67)	0.065
	*Culex* spp.	Control	25	288	2.37 (1.35–4.17)	1	0	
		TF-sisal ribbon	25	89	0.56 (0.29–1.06)	0.23 (0.12–0.43)	0.77 (0.56–0.87)	< 0.001

*n* total number of mosquito collected, *CI* confidence interval, *PP* percentage protection, *RR* relative rate, *TF* transfluthrin, 1 and 0 references

**Table 4:** Comparison of nightly outdoor biting per person between houses with or without transfluthrin-treated chairs or ribbons (wet season)

Settings	Species	Treatment	Nights	n	Adjusted-mean (95% CI)	RR (95% CI)	PP (95% CI)	P-value
Outdoor peri-domestic space	*Anopheles arabiensis*	Control	32	1116	5.71 (4.89–6.67)	1	0	
		One TF-chair	32	308	1.42 (1.17–1.72)	0.25 (0.20–0.31)	0.75 (0.69–0.79)	< 0.001
		Two TF-chairs	32	189	0.86 (0.69–1.07)	0.15 (0.12–0.18)	0.85 (0.81–0.88)	< 0.001
		TF-treated ribbon	32	273	1.32 (1.08–1.60)	0.23 (0.18–0.28)	0.77 (0.71–0.81)	< 0.001
	*Culex* spp.	Control	32	4142	21.78 (18.11–26.18)	1	0	
		One TF-chair	32	2598	13.17 (10.93–15.86)	0.60 (0.53–0.68)	0.40 (0.31–0.47)	< 0.001
		Two TF-chairs	32	2216	10.68 (8.85–12.87)	0.49 (0.43–0.56)	0.51 (0.44–0.57)	< 0.001
		TF-treated ribbon	32	2794	13.93 (11.56–16.78)	0.64 (0.56–0.72)	0.36 (0.27–0.44)	< 0.001
Inside outdoor kitchen enclosure	*Anopheles arabiensis*	Control	32	68	Low catches			
		TF-sisal ribbon	32	24	Low catches			
	*Culex* spp.	Control	32	302	0.49 (0.31–0.78)	1	0	
		TF-sisal ribbon	32	172	0.26 (0.15–0.43)	0.52 (0.32–0.86)	0.48 (0.13–0.67)	0.011

*n* total number of mosquito collected, *CI* confidence interval, *PP* percentage protection, *RR* relative rate, *TF* transfluthrin, 1 and 0 references

Fitting the transfluthrin-treated hessian ribbons around the outdoor kitchens reduced outdoor-biting *An. arabiensis* by 81% in the area immediately outside this kitchen enclosure (RR = 0.19, 95% CI 0.16–0.24, *P *< 0.001), and by 43% (RR = 0.57, CI 0.32–1.03, *P *= 0.065) inside the enclosures in the dry season. In the wet season, transfluthrin-treated hessian ribbons reduced outdoor-biting *An. arabiensis* by 77% in the area immediately outside this kitchen enclosure (RR = 0.23, 95% CI 0.18–0.28, *P *< 0.001). The ribbons also reduced outdoor-biting *Culex* by 68% (RR = 0.32, CI 0.24–0.43, *P *< 0.001) near the enclosures and by 77% (RR = 0.23, CI 0.12–0.43, *P *< 0.001) within the enclosures in the dry season. In the wet season, the ribbons also reduced outdoor-biting *Culex* by 36% (RR = 0.64, CI 0.56–0.72, *P *< 0.001) near the enclosures and by 48% (RR = 0.52, CI 0.32–0.86, *P *< 0.001) within the enclosures.

### Mortality of field-collected or laboratory-reared mosquitoes exposed to transfluthrin-treated chairs

Findings on induced mortality of mosquitoes exposed to transfluthrin-treated chairs are summarized in Table 5. When field-collected *An. arabiensis* females and *An. funestus* females were exposed underneath the transfluthrin-treated chairs, 99.4% and 100% of them died within 24 h, respectively. All (100%) of the laboratory-reared *An. arabiensis* or laboratory-reared *Ae. aegypti* mosquitoes exposed also died when exposed underneath the transfluthrin-treated chairs. Mortality of the mosquitoes exposed to untreated chairs however remained low (5.2% for field-collected *An. arabiensis*, 0.0% for field-collected *An. funestus*, 0.1% for laboratory-reared *An. arabiensis* and 1.1% for laboratory-reared *Ae. aegypti*).

**Table 5:** Comparison of induced mortality to mosquitoes exposed to house with or without transfluthrin-treated chairs

Settings	Species	Treatment	Days	Exposed	Dead 24 h	Mortality (%)
Wild mosquitoes	*Anopheles arabiensis*	Control	10	1142	60	5.2
		TF-treated chair	10	1140	1134	99.4
	*Anopheles funestus*	Control	5	490	0	0
		TF-treated chair	5	490	490	100
Lab-reared mosquitoes	*Anopheles arabiensis*	Control	9	860	10	1.1
		TF-treated chair	9	860	860	100
	*Aedes aegypti*	Control	9	900	3	0.3
		TF-treated chair	9	900	900	100

*TF* transfluthrin

### Insecticide resistance status of mosquitoes in a study area

Results of the WHO resistance tests are summarized in Table 6. The field populations of *An. arabiensis* were fully susceptible to bendiocarb (100% mortality), pirimiphos methyl (100% mortality) and DDT (98.8% mortality). However, they were resistant to both permethrin (94.7% mortality) and deltamethrin (80.3% mortality).

**Table 6:** Show insecticide resistant status in *Anopheles arabiensis* mosquitoes to difference insecticides at Lupiro village

Insecticide tested	Mosquito species tested	Percentage mortality (%)	Resistance status
Bendiocarb	*Anopheles arabiensis*	100	Susceptible
Pirimiphos-methyl	*Anopheles arabiensis*	100	Susceptible
DDT	*Anopheles arabiensis*	98.8	Susceptible
Permethrin	*Anopheles arabiensis*	94.7	Resistant (after confirmation)
Deltamethrin	*Anopheles arabiensis*	80.3	Resistant

*DDT* dichlorodiphenyltrichloroethane

## Discussion

Several studies in tropical settings have documented that many people stay active outdoors in early evenings before they go indoors and then sleep under bed nets [14, 16, 17]. Those studies also characterized the actual activities that people were involved in outdoors. To our knowledge, this current study is the first to characterize the peri-domestic spaces used by household members in a malaria-endemic setting for various outdoor activities.

The key finding was that most houses had active peri-domestic spaces (veranda extensions, open general areas and makeshift kitchens) where household members performed different activities, usually unprotected from potentially-infectious mosquitoes before they went indoors. In some of the peri-domestic spaces, residents constructed structures for cooking, eating and socializing, but these too were often open and not protective against mosquito bites (Fig. 3b).

The study also demonstrated that the two simple interventions evaluated, i.e. transfluthrin-emanating chairs and ribbons both considerably reduced outdoor-biting by the important residual malaria vector, *An. arabiensis*. Furthermore, mosquitoes exposed to the chairs were killed rapidly, indicating that the interventions could offer not just personal or household protection, but also communal protection through mass killing effect, by reducing mosquito density, survival and malaria sporozoite infection prevalence [37].

More than half the households surveyed had veranda extensions with roofed enclosures, mostly used for resting, cooking and eating. All these structures provide opportunities for mounting simple interventions in these spaces such as physical screening and complementary chemical measures like these transfluthrin emanator formats and turning them into mosquito proof areas as they are predominantly used for early-evening human activities, notably resting, cooking and eating.

The findings that transfluthrin-emanating chairs provided useful levels of protection against *An. arabiensis* and *Culex* spp. corroborate previous observations with other prototypes in outdoor bars [33]. Even though the prototype (chair) used in this study differs to those used in previous studies (decoration) [33], it emphasizes the potential of these technologies for outdoor protection in such communities. Further research should therefore focus on improvement of the prototypes and optimization of the treatments.

Outdoor kitchens were commonly used for cooking in early evening, and were among the commonest constructed spaces identified in households, regardless of whether they had verandas or not. Early-evening cooking within this space coincides with peak hours of mosquito bites [54], amplifying the likelihood of malaria transmission in these spaces. In this study, the high levels of protection provided against *An. arabiensis* by the repellent-treated hessian ribbons around these outdoor kitchens is, therefore, encouraging and consistent with previous studies [34], which demonstrated that transfluthrin-treated hessian ribbons protected non-users against *An. arabiensis* sitting within radius of 5 metres. More recently, transfluthrin-treated ribbons fitted to the eaves of houses prevented both indoor and outdoor-biting mosquitoes [31, 36, 37]. Since the increase in temperature also increases the rate of transfluthrin evaporation, the cooking activity within the kitchen may have increased insecticidal activity of transfluthrin. The effect of temperature was also well described by Ogoma et al. [34]. This high level of protection provided against *An. arabiensis* by the ribbons may have been positively influenced by cooking activities within these enclosures.

In addition to the substantial protection against *An. arabiensis* demonstrated in the areas immediately outside the ribbon-fitted kitchen, the catches by CDC light traps placed within the kitchens are reduced, albeit more modestly. This modest reduction may be due to the use of CDC light traps in these open spaces, which may have resulted in exaggerated catches of mosquitoes attracted by the light bulb in the traps. It may also be due to the smoke produced from these kitchens, which may have confounded the results observed on *An. arabiensis*. Interestingly, this emanator prototype provided much more satisfactory protection against nuisance-causing *Culex* spp. within the kitchens based on the same CDC light trap catches. It is not clear why such significant reductions observed for *Culex* spp. were not observed for *An. arabiensis*, but it is nevertheless encouraging that reduced *Culex* spp. densities should motivate user acceptance. It is also encouraging that these observations are also broadly consistent with previous studies [31, 32] demonstrating that outdoor use of transfluthrin-treated hessian provided more than 90% protection against both *An. gambiae* s.l. and *Culex* spp. mosquitoes [31, 32].

Pyrethroid-treated nets divert host-seeking mosquitoes from humans or kill the mosquitoes attempting to feed on the protected persons [55, 56]. With these modes of action, pyrethroid-treated nets not only provide personal protection (to users), but also communal protection (to both users and non-users) by suppressing vectors population through the mass killing effect [57, 58]. Transfluthrin, used to treat the hessian mats fitted underneath the chairs induced high mortality on caged mosquitoes exposed underneath the experimental chairs (100% in most cases). This implies that the chairs may not only provide personal protection, but also community benefit through mass-killing of mosquitoes, even without the mosquitoes making contact with treated surfaces. This effect was particularly important since the field-collected mosquitoes were from villages where *Anopheles* populations were pyrethroid-resistant (Table 6).

To date, there is no literature which explains the best exposure time for mosquitoes in transfluthrin-treated material that achieves 50% mortality. However, Ogoma et al. [38, 59] demonstrated that even short exposures of 15 min reduced mosquito blood feeding significantly. In this current study, the selection of exposure time was based on what period a particular mosquito species is active. For the day-biting mosquitoes, a day-time exposure was selected and for night-time biting species a night-time exposure was selected.

Even though excito-repellency effects maximize person protection by chasing mosquitoes away, it may attenuate more important mass killing effects by deterring mosquitoes from making fatal contact with lethal doses of the repellent insecticide itself or with complementary solid-phase insecticides applied as LLINs or IRS [60–62]. However, these observations of mortality amongst wild malaria vectors exposed to transfluthrin suggest that mass population suppression could be achieved even without mosquitoes necessarily touching treated surfaces. It is also encouraging that Ogoma et al. [34] demonstrated that transfluthrin-treated emanator provided more than 90% biting reduction against *An. arabiensis* without any obvious diversion to non-users [34]. Another study by Ogoma et al. [38] also observed that transfluthrin-treated coils could protect non-users within 20 m radius. More recently, Mwanga et al. demonstrated that transfluthrin-treated ribbons fitted to the eave gaps of houses protected volunteers both inside and outside the houses [37].

The spread of pyrethroid resistance in malaria vectors clearly compromises ongoing control and elimination efforts [63–65]. This is a key concern since transfluthrin is also a pyrethroid. It is however encouraging that transfluthrin-based interventions tested here killed almost 100% of the wild-caught *An. arabiensis* and *An. funestus* exposed to emanated vapour from the chairs, even though local populations of both species are clearly resistant to the conventional solid-phase pyrethoids used for LLINs and IRS [41]. It was surprising that transfluthrin, a pyrethroid, was still efficacious against pyrethroid-resistant malaria mosquitoes. However, given that there is no standard resistance test against transfluthrin, it is difficult to explain as to why transfluthrin demonstrated such high mortality. One possible explanation is the long exposure of up to 12 h underneath the transfluthrin-treated chairs. Tests with PBO have established that the resistance in this area is of metabolic nature, thus it may be helpful that these new interventions are considered as complementary to other interventions, e.g. IRS or LLINs using active ingredients not affected by this form of resistance.

**Fig. 4:**
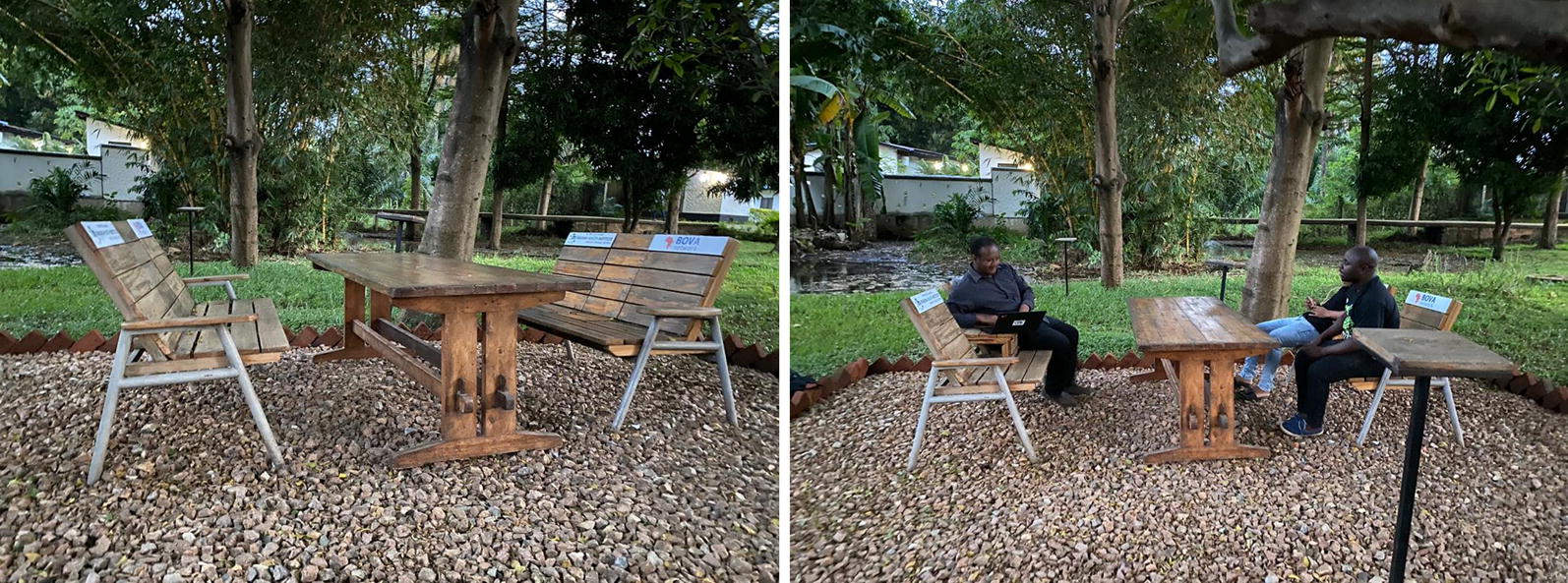
Picture of the first mosquito-free zone established at Ifakara Health Institute in January 2020. The chairs have transfluthrin-treated hessian mats underneath, but are layered with plastic sheeting to prevent rainfall and user contact

Usage of chairs cut across different settings, such as normal households, public places, official surroundings used for resting after working hours (Fig. 4). Based on this information, the use of transfluthrin-treated chairs may be rolled out as a complementary vector control strategy even during dengue fever outbreak.

One important limitation of this study was that caged mosquitoes were placed underneath the transfluthrin-treated chairs for 12 h. This long-time exposure may well greatly exceed true exposure levels in the field, where mosquitoes can freely fly around and way upon encountering airborne insecticide. Nonetheless, since transfluthrin effects are vapor-mediated, this initial attempt to quantify possible lethal modes of action is encouraging and offers a basis for future improvements in study designs for developing and evaluating these technologies.

## Conclusions

Most houses in this rural African context had well-used peri-domestic spaces (veranda extensions, makeshift kitchens and completely open spaces) where members performed different activities before bed time, usually unprotected from potentially-infectious mosquitoes before they went indoors. Both the transfluthrin-emanating chairs and ribbons reduced outdoor exposure to biting malaria vectors in these peri-domestic spaces and also caused significant mortality of caged, field collected malaria vector mosquitoes. The two emanator prototypes still require additional improvements, optimizations and assessments in future studies, but they could potentially constitute new options for outdoor malaria prevention to complement LLINs and IRS in areas where peri-domestic human activities are common.

## Data Availability

Not applicable.

## Abbreviations

CDC: Center of disease control
CI: Confidence interval
GLMM: Generalized linear mixed effects model
IRS: Indoor residual spraying
IHI: Ifakara Health Institute
IRB: Institutional Review Board
LLINs: Long-lasting insecticidal nets
NIMR: National Institute for Medical Research
PCR: Polymerase chain reaction
RR: Relative rate
